# Efficacy and safety of RGB-02, a pegfilgrastim biosimilar to prevent chemotherapy-induced neutropenia: results of a randomized, double-blind phase III clinical study vs. reference pegfilgrastim in patients with breast cancer receiving chemotherapy

**DOI:** 10.1186/s12885-019-5329-6

**Published:** 2019-02-06

**Authors:** Zsuzsanna Kahan, Daniela Grecea, Martin Smakal, Sergei Tjulandin, Igor Bondarenko, Luca Perjesi, Andras Illes, Karoly Horvat-Karajz, Ildiko Aradi

**Affiliations:** 1 Department of Oncotherapy, University of Szeged, Korányi Fasor 12, Szeged, 6720 Hungary; 2 Institutul Oncologic Prof. Dr. I. Chiricuta, Republicii Bulevardul 34-36, 400015 Cluj-Napoca, Romania; 3 Nemocnice Horovice, K nemocnici 1106, 268 01 Horovice, Czech Republic; 4 Russian Cancer Research Center of the Russian Academy of Medical Sciences, Kashirskoye Shosse 24, Moscow, Russia 115478; 5 Department of Oncology and Medical Radiology, Dnipropetrovsk Medical Academy, Vernadsky str. 9, Dnipropetrovsk, 49044 Ukraine; 6 Gedeon Richter Plc, Budapest, Hungary; Gyömröi út 19-21, Budapest, 1103 Hungary

**Keywords:** Pegfilgrastim, Biosimilar, Chemotherapy-induced neutropenia, RGB-02, Clinical study, Breast Cancer, Therapeutic equivalence

## Abstract

**Background:**

Treatment with recombinant human granulocyte-colony stimulating factor (G-CSF) is accepted standard for prevention of chemotherapy-induced neutropenia. RGB-02 (Gedeon Richter) is a proposed biosimilar to pegylated G-CSF (Neulasta®, Amgen) with sustained release properties. This is a randomized, comparative, double-blind, multicenter study to evaluate efficacy and safety of RGB-02 in breast cancer patients receiving cytotoxic regimen.

**Methods:**

Two hundred thirty-nine women presenting with breast cancer were randomized to RGB-02 (*n* = 121) and the reference product (*n* = 118). All patients received up to 6 cycles of docetaxel/doxorubicin chemotherapy combination and a once-per-cycle injection of a fixed 6 mg dose of pegfilgrastim. Primary endpoint was the duration of severe neutropenia (ANC < 0.5 × 10^9^/L) in Cycle 1 (2-sided CI 95%). Secondary endpoints included incidence and duration of severe neutropenia (in cycles 2–4), incidence of febrile neutropenia, time to ANC recovery, depth of ANC nadir, and safety outcomes.

**Results:**

The mean duration of severe neutropenia in Cycle 1 was 1.7 (RGB-02) and 1.6 days (reference), with a difference (LS Mean) of 0.1 days (95% CI -0.2, 0.4). Equivalence could be established as the CI for the difference in LS Mean lay entirely within the pre-defined range of ±1 day. This positive result was supported by the analysis of secondary endpoints, which also revealed no clinical meaningful differences. Safety profiles were comparable between groups. No neutralizing antibodies against pegfilgrastim were identified.

**Conclusions:**

Treatment equivalence in reducing the duration of chemotherapy induced neutropenia between RGB-02 and Neulasta® could be demonstrated. Similar efficacy and safety profiles of the once-per-cycle administration of RGB-02 and the pegfilgrastim reference were demonstrated.

**Trial registration:**

The trial was registered prospectively, prior to study initiation. EudraCT number (2013–003166-14). The date of registration was 12 July, 2013.

**Electronic supplementary material:**

The online version of this article (10.1186/s12885-019-5329-6) contains supplementary material, which is available to authorized users.

## Background

RGB-02 (Gedeon Richter) is a proposed biosimilar medicinal product to Neulasta® (Amgen) which has been approved in the European Union (EU) in 2002 and is commonly used to decrease the duration of chemotherapy-induced neutropenia and to reduce the probability of febrile neutropenic episodes in patients treated with cytotoxic chemotherapy for malignancy. The active substance of RGB-02 is pegfilgrastim, the pegylated form of filgrastim, which constitutes a covalent conjugate of recombinant human granulocyte-colony stimulating factor (G-CSF) with a single 20 kDa polyethylene glycol (PEG) [1]. Filgrastim, approved in 1991 is a non-glycosylated protein with a methionine group attached to the human amino acid sequence and is produced by recombinant-DNA technology in *Escherichia coli*. Filgrastim is eliminated from the circulation by rapid renal clearance therefore requires daily administration in each chemotherapy cycle [2]. In contrast, pegfilgrastim is mainly eliminated by neutrophil-mediated clearance, resulting in a long serum half-life and therefore allows a single administration per chemotherapy-cycle [3, 4]. This clear advantage over filgrastim which has to be administered daily, leads to a better patient compliance and results in improved clinical outcomes [5–7].

Since filgrastim biosimilars referring to the reference product Neupogen® are authorized in Europe since 2009 and in the US since 2015, no biosimilars of pegfilgrastim are approved yet although some compounds are in different stages of development [8–10]. The development of biosimilars is regulated through specific guidelines to guarantee similarity with the reference product in quality, pharmacokinetics, −dynamics, and clinical efficacy as well as the safety profile [11–14].

We are reporting here the results of a clinical study comparing efficacy and safety of the proposed biosimilar RGB-02 with the reference compound Neulasta® (hereinafter referred to as reference). This phase III study was designed as prospective, randomized, double-blind, parallel-group, multinational, multicentric trial to demonstrate confirmatory equivalence in terms of pharmacodynamic and clinical parameters in breast cancer patients receiving docetaxel/doxorubicin as myelosuppressive chemotherapy combination.

## Methods

Between January 2014 and April 2015, we included 238 patients with breast cancer in 35 centers (located in Hungary, Romania, Czech Republic, Bulgaria, Croatia, Serbia, Russia, Ukraine) receiving chemotherapy on Day 1 of each cycle with 60 mg/m^2^ doxorubicin infusion followed by 75 mg/m^2^ docetaxel (EudraCT number 2013–003166-14). The study protocol considered all relevant regulatory and scientific guidelines [15–17] and was approved by all involved national regulatory authorities and ethics committees. The performance and supervision of this trial followed the principles of Good Clinical Practice (GCP) as laid down in ICH E 6 [18]. No interim analysis was performed and no Data Monitoring Committee operated in this study.

### Patients

The study population included chemotherapy-naïve women ≥18 and ≤ 65 years of age with invasive breast cancer (Stage IIB and III) appropriate for combined treatment with doxorubicin/ docetaxel in the neo−/adjuvant treatment setting. Additional inclusion criteria were Eastern Cooperative Oncology Group (ECOG) performance status 0 or 1 and adequate bone marrow function, as indicated by absolute neutrophil count (ANC) ≥ 1.5 × 10^9^/L, platelet count ≥100 × 10^9^/L, and Hemoglobin > 8 g/dL; adequate renal and hepatic function with an estimated creatinine clearance ≥50 mL/min (Cockcroft-Gault method), bilirubin, aspartate transaminase, alanine transaminase < 1.5 x upper limit of normal (ULN), and Alkaline phosphatase < 2.5 x ULN. Women with childbearing potential had to present a negative urine pregnancy test and had to agree to use 2 reliable methods of contraception during treatment period and for 3 months thereafter. Written informed consent had to be given prior to any study-related procedure. Main exclusion criteria were any other malignancy within 5 years prior to randomization, with the exception of cervical carcinoma in situ, non-melanoma skin cancer, or superficial bladder tumors (Ta, Tis, or T1) that had been successfully and curatively treated; active infection or systemic anti-infective treatment, radiation therapy within 4 weeks prior to randomization; past exposure to any G-CSFs; concurrent anti-cancer therapy, concomitant treatment with bisphosphonates; prior bone marrow or stem cell transplantation; history or presence of sickle cell disease, and significant cardiovascular disease (caution especially for doxorubicin).

### Randomization and study treatment

Eligible patients were randomly assigned to receive the study drugs (6 mg s.c. of either RGB-02 or reference) in a 1:1 ratio via an interactive voice/web response system (IXRS). On day 1 of each treatment cycle, all patients received 60 mg/m^2^ doxorubicin followed 1 h later by an intravenous infusion of 75 mg/m^2^ docetaxel. Chemotherapy was to be repeated every 3 weeks for up to 6 cycles. Patients were dosed with the study drugs approximately 24 h after chemotherapy was initiated for each cycle.

### Study procedures

The study design was set up in a double blind fashion for the first 2 treatment cycles to demonstrate confirmatory efficacy followed by an open-label safety assessment during treatment the subsequent cycles. Patients in the reference arm were switched to open-label RGB-02 starting with cycle 3. After the initial 3-weeks-screening period and baseline visits, patients were scheduled for 4 chemotherapy cycles (and 2 additional if deemed necessary) each requiring 12 study visits within 3 weeks followed by a final safety assessment 6 months after treatment start. Pre-defined hematology blood samplings to determine the absolute neutrophil count for efficacy assessment were performed on Days − 1, 1, 2, 3, daily from Day 5 to Day10 and on Days 14 and 18. All patients underwent a baseline clinical examination that included physical examination, pregnancy testing, safety monitoring including hematology data (Hemoglobin; WBC count, lymphocytes (absolute), neutrophils (absolute), platelets); testing of hepatic function (aspartate aminotransferase, alanine aminotransferase, and bilirubin), lactate dehydrogenase, albumin, blood urea nitrogen, uric acid, creatinine clearance.

Blood sampling for immunogenicity assessments of pegfilgrastim was performed at Day − 1 of Cycles 1, 3 and 4, before any study treatment was administered and at the end of Cycle 4 and at follow-up. A stepwise approach was established for immunogenicity assessment, namely all samples were analyzed with screening assays for anti-RGB-02 and anti Neulasta® antibodies. The samples assessed positive with the screening assays were to be analyzed with confirmatory assays and the confirmed positive samples would have been analyzed with a cell-based neutralizing assay*.* Immunogenicity assays used were developed and validated in line with the applicable guidelines and recommendations [14, 19, 20].

#### Study outcomes

The primary efficacy variable was the mean duration of severe neutropenia (DSN) in chemotherapy cycle 1 whereas DSN was defined as the number of consecutive days in which a patient had an ANC < 0.5 × 10^9^/L.

Other secondary outcomes comprised the duration of severe neutropenia (ANC < 0.5 × 10^9^/L) in Cycles 2, 3 and 4, the incidence of severe neutropenia as well as febrile neutropenia in Cycles 1 and 2, time to ANC recovery and the depth of ANC nadir in Cycles 1 and 2.

The incidence of febrile neutropenia was based on the ESMO definition of an oral temperature > 38.5 °C or 2 consecutive readings of > 38.0 °C for 2 h and an ANC < 0.5 × 10^9^/L (or expected to fall below 0.5 × 10^9^/L), whereas the overall incidence of febrile neutropenia included beyond the ESMO definition any administration of systemic antibiotics if treatment with the antibiotics was commenced while the ANC was under 1.5 × 10^9^/L [21].

The safety assessment was performed by continuously evaluating adverse events according Common Terminology Criteria for Adverse Events (CTCAE), Version 4.03. Further safety assessments included physical examinations with special regard to the site of pegfilgrastim injection, vital signs, ECG, pulse oximetry and laboratory tests conducted at baseline and at defined time points post dose. Hematology parameters were assessed on Days 1, 3, 5–10, 14 and 18, while the timing of other lab tests varied depending on the parameter. A follow-up visit was performed 6 months after individual study start.

### Statistical analysis

The sample size of 111 evaluable patients per treatment arm was determined based on an equivalence test of means using two 1-sided tests on data from a parallel-group design in order to achieve 90% power at 5% significance level when the true difference between the means was assumed to be 0.25, the standard deviation (std) was assumed to be 1.70, and the equivalence limits were − 1.00 and 1.00 days.

The primary efficacy variable was the duration of severe neutropenia, defined as ANC < 0.5 × 10^9^/L, in the first cycle of chemotherapy. The difference in mean duration of severe neutropenia between the 2 treatment arms and the 2-sided 95% confidence interval (CI) for the difference between means was calculated using an analysis of covariance (ANCOVA) model with treatment, country, chemotherapy treatment setting (neoadjuvant or adjuvant) as factors, and baseline ANC value (value at Day − 1, Cycle 1) as covariate in the model.

If the upper limit of the 95% CI for the difference in means was ≤1 day and the lower bound of the CI for the difference in means was ≥ − 1 day, then the means in the 2 arms were to be considered equivalent. A similar analysis was performed for Cycle 2.

The duration of severe neutropenia in Cycles 3 and 4 as well as the depth of ANC nadir in Cycles 1 and 2 were summarized using descriptive statistics. An ANCOVA analysis was also performed for the difference in depth of ANC nadir.

The difference in the incidence of patients with febrile neutropenia in Cycles 1 and 2 between the 2 treatment arms with associated 95% CI was presented. Time to ANC recovery in Cycles 1 and 2 was analyzed using Kaplan-Meier life table methods. The analyses were performed using the protocol definition for ANC recovery (number of days from any ANC value < 0.5 × 10^9^/L to ANC ≥ 2 × 10^9^/L) and repeated for the alternative definition (number of days from the date of the lowest measured ANC value to ANC ≥ 2 × 10^9^/L). The primary data set for efficacy analysis was the per-protocol (PP) population; the full analysis set (FAS) was analysed in addition for demonstrating robustness of data. All patients who received at least one dose of a study medication were included in the safety analysis. Safety variables were summarized by treatment arm. All statistical analyses were performed using SAS 9.2 software.

## Results

**Fig. 1:**
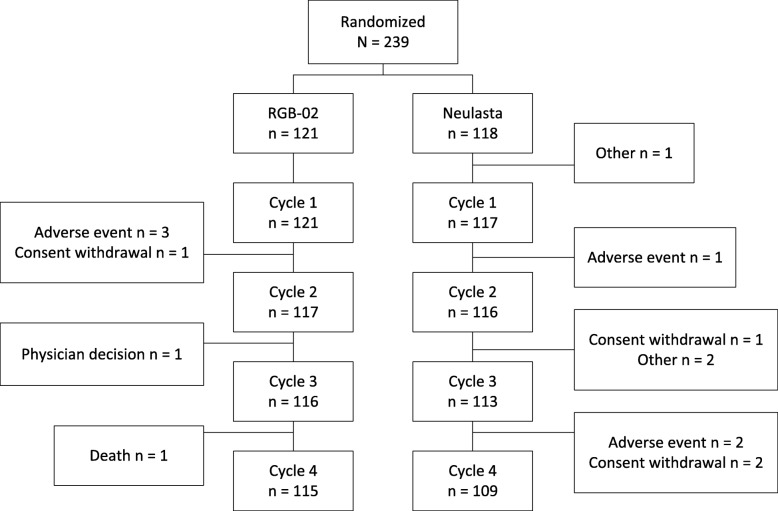
Patient flow. Note: One of the adverse events (AEs) leading to withdrawal during Cycle 1 in the RGB-02 arm resulted in death after patient withdrawal

A total of 239 patients were randomized (1:1) to receive either RGB-02 or the reference product prior to undergoing chemotherapy at 35 study centers. One patient was excluded (this patient randomized to the comparator arm did not meet inclusion criteria and discontinued without receiving study medication) leaving 238 patients in the FAS (Full analysis set) population, of whom 121 received RGB-02 and 117 received reference product. The FAS collective also served as safety data set, detailed patient disposition is listed in Fig. 1.

There were generally no differences in patient characteristics at baseline between groups (Table 1). Stage of breast cancer was IIB in 47.9% of patients and III in 50.8% of patients, with no differences observed between study arms. Adjuvant chemotherapy setting was more common than neoadjuvant in the RGB-02 arm (57.9 and 42.1%, respectively), slightly different - but not clinically meaningful - to the comparator arm (adjuvant: 50.4%, neoadjuvant: 49.6%). Both groups were comparable regarding medical history, concomitant medication, and surgical interventions for the underlying breast cancer.

**Table 1:** Patient characteristics: Full Analysis Set

Variable	RGB-02 (*N* = 121)	Reference (*N* = 117)	Total (*N* = 238)
Race [n (%)]			
White	120 (99.2)	117 (100)	237 (99.6)
Asian	1 (0.8)	0	1 (0.4)
Age (years)			
Mean (std)	51.0 (8.20)	51.2 (9.56)	51.1 (8.88)
Weight (kg)			
Mean (std)	72.17 (14.049)	74.83 (15.240)	73.48 (14.676)
Height (cm)			
Mean (std)	163.3 (6.58)	163.5 (6.29)	163.4 (6.43)
BSA (m^2^)			
Mean (std)	1.791 (0.1718)	1.815 (0.1812)	1.803 (0.1765)
Stage of disease [n (%)]			
Stage IIB	58 (47.9)	56 (47.9)	114 (47.9)
Stage III	61 (50.4)	60 (51.3)	121 (50.8)
Chemotherapy treatment [n (%)]			
Neoadjuvant	51 (42.1)	58 (49.6)	109 (45.8)
Adjuvant	70 (57.9)	59 (50.4)	129 (54.2)

*BSA* body surface area; std.standard deviation

### Primary efficacy endpoint

Therapeutic equivalence could be demonstrated between RGB-02 and the reference pegfilgrastim with 95% CIs within the predefined margins of ±1 day confirming equivalence.

The mean duration of severe neutropenia (DSN, defined as the number of days from the first ANC value < 0.5 × 10^9^/L until increasing back ≥0.5 × 10^9^/L) in the PP collective during cycle 1 was comparable in the RGB-02 arm (1.7 ± 1.14 days) and the reference arm (1.6 ± 1.31 days). The LS Means (95% CI) were 1.5 (1.2, 1.8) and 1.4 (1.1, 1.7) days, respectively. The LS Mean for the difference in duration of severe neutropenia was 0.1 days (95% CI: -0.2, 0.4), identical to that observed in the FAS population. In cycle 2 the DSN declined equally to 0.7 days for both compounds (Table 2). The mean duration of severe neutropenia in Cycles 3 and 4, after patients in the reference arm switched to RGB-02, was < 1 day in the RGB-02 arm (0.9 days) and the reference arm switched to RGB-02 (0.6 days), indicating that the switch from reference to RGB-02 treatment did not increase the patient’s risk to develop longer lasting grade 4 neutropenia.

**Table 2:** Duration of Severe Neutropenia

	RGB-02	Reference	Difference(RGB-02 - Reference)
Cycle 1, PP population			
n	112	111	
Mean (SD)	1.7 (1.14)	1.6 (1.31)	
Least squares mean (95% CI)	1.5 (1.2, 1.8)	1.4 (1.1, 1.7)	0.1 (−0.2, 0.4)
Cycle 1, FAS			
n	121	117	
Mean (SD)	1.8 (1.28)	1.7 (1.45)	
Least squares mean (95% CI)	1.6 (1.3, 1.9)	1.4 (1.1, 1.7)	0.1 (−0.2, 0.4)
Cycle 2, PP population			
n	111	100	
Mean (SD)	0.7 (0.81)	0.7 (0.97)	
Least squares mean (95% CI)	0.7 (0.4, 0.9)	0.6 (0.4, 0.8)	0.1 (−0.2, 0.3)
Cycle 2, FAS			
n	117	116	
Mean (SD)	0.7 (0.81)	0.9 (1.31)	
Least squares mean (95% CI)	0.5 (0.3, 0.8)	0.8 (0.5, 1.0)	-0.2 (−0.5, 0.1)

The similarity of results in the primary efficacy variable between the PP population and the FAS further supports the observed equivalent effect and robustness of data.

### Secondary efficacy endpoints

Neither statistically significant nor clinically relevant differences were detected in secondary endpoints between treatment groups.

**Fig. 2:**
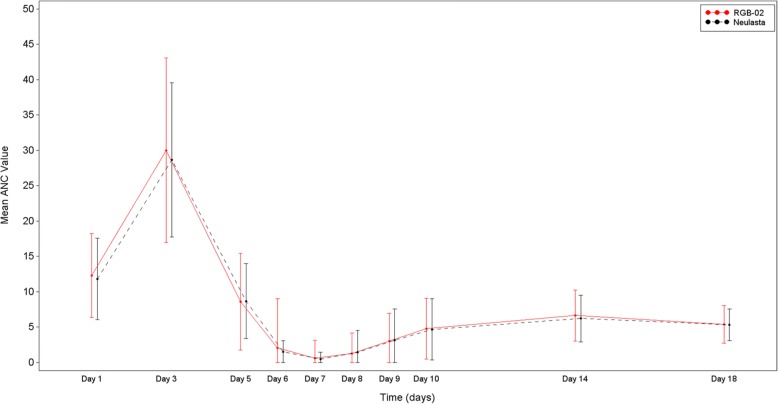
Mean ANC Values by Day and Treatment Arm – Cycle 1

The mean daily ANC values for both groups in Cycle 1 were almost identical (Fig. 2).

Most patients experienced severe neutropenia (defined as ANC < 0.5 × 10^9^/L) during cycle 1. The incidence of severe neutropenia decreased in cycle 2 compared to cycle 1 in both treatment groups with no statistical significant differences, for RGB-02 from 84.6% (99 patients) to 54.1% (60 patients) and for the comparator groups from 77.0% (87 patients) to 43.7% (45 patients) (Table 3).

**Table 3:** Incidence of Severe Neutropenia

	RGB-02	Reference	Difference(RGB-02 - Reference)
Cycle 1, PP population			
n	117	113	
n (%) with severe neutropenia	99 (84.6)	87 (77.0)	
Proportion (95% CI) with severe neutropenia	0.846 (0.768, 0.906)	0.770 (0.681, 0.844)	0.076 (−0.055, 0.204)
Cycle 2, PP population			
n	111	103	
n (%) with severe neutropenia	60 (54.1)	45 (43.7)	
Proportion (95% CI) with severe neutropenia	0.541(0.443, 0.636)	0.437(0.339, 0.538)	0.104(−0.031, 0.236)

During Cycle 1, the observed incidence and the overall incidence of febrile neutropenia including cases meeting the ESMO criteria and those who started systemic antibiotic treatment was similar in both groups with 5 patients [4.3%] and 10 patients [8.5%]) in the RGB-02 arm and 4 patients [3.5%] and 8 patients [7.1%]) in the reference arm. During Cycle 2, no febrile neutropenia was observed in any of the treatment arm except one overall febrile neutropenia case in the RGB-02 group (Table 4).

**Table 4:** Observed Incidence of Febrile Neutropenia

	RGB-02	Reference	Difference(RGB-02 - Reference)
Cycle 1, PP population			
n	117	113	
n (%) with febrile neutropenia	5 (4.3)	4 (3.5)	
Proportion (95% CI) with febrile neutropenia	0.043 (0.014, 0.097)	0.035(0.010, 0.088)	0.007(−0.123, 0.137)
Cycle 2, PP population			
n	111	103	
n (%) with febrile neutropenia	0	0	

There were no significant differences between groups regarding mean time to ANC recovery (defined as recovery from any ANC value < 0.5 × 10^9^/L to ANC ≥ 2 × 10^9^/L). During Cycle 1, mean time to recovery was 3.4 ± 1.84 days in the RGB-02 arm and 3.7 ± 1.88 days in the reference arm; during Cycle 2, mean time to recovery was 2.8 ± 1.09 days and 3.4 ± 2.11 days, respectively. The recovery from the date of the lowest measured ANC value was comparable between groups, too (Fig. 2). Also, no clinically meaningful difference was found when comparing the mean depth of ANC nadir in Cycle 1 and 2.

### Safety

When analysing the safety population one has to take into account that the reference drug was given only for the double-blind period of the first 2 Cycles. All patients from that group received thereafter RGB-02 for cycles 3–6. The safety population for the comparative safety analysis (Table 5) comprised 238 women (RGB-02 = 121, reference = 117) whereas altogether 234 women received 995 doses of RGB-02 throughout the course of the study. In total, 204/234 (87.2%) patients treated with RGB-02 had at least 1 adverse event (AE).

**Table 5:** Overall Frequency of Adverse Events

	RGB-02(*N* = 121) n (%)	Reference(*N* = 117) n (%)
Number (%) of patients with:		
Any AE	111 (91.7)	113 (96.6)
Any Grade ≥ 3 AE	23 (19.0)	18 (15.4)
Any AE related to IMP	26 (21.5)	32 (27.4)
Any serious AE	13 (10.7)	12 (10.3)
Any IMP-related serious AE	0	0
Any AE leading to withdrawal	2 (1.7)	4 (3.4)
Any IMP-related AE leading to withdrawal	0	0
Any AE with an outcome of death	2 (1.7)	0
Any IMP-related AE with an outcome of death	0	0
Any injection site reaction AE	2 (1.7)	2 (1.7)

The cumulative incidence of adverse events was similar between both groups.

During Cycles 1 and 2, 80.2% treatment-emergent adverse events (TEAE) were reported in the RGB-02 arm (*n* = 97) compared to 93.2% in the reference arm (*n* = 109). Similarly, the number of patients with IMP-related AEs was marginally lower in the RGB-02 arm (17 patients [14.0%]) compared to the reference arm (27 patients [23.1%]). The most frequent pegfilgrastim-related AE was bone pain, slightly less frequently reported in the RGB-02 arm (14 patients [11.6%]) compared to the reference arm (20 patients [17.1%]).Other musculoskeletal and connective tissue disorders included arthralgia in the RGB-02 arm (2 patients [1.7%]. Myalgia (3 patients [2.6%], pain in extremity and spinal pain (2 patients [1.7%] each) were reported in the reference arm only.

Serious adverse events (SAE) were reported during the double-blind period at Cycles 1 and 2 in 8.3% of the RGB-02 arm and 6.8% of the reference arm cases, none of them related to pegfilgrastim (Table 6). The most frequent SAE was febrile neutropenia: 4.1% of patients in the RGB-02 arm and 5.1% of patients in the reference arm. There were 2 deaths reported in the RGB-02 arm, none of them were related to the investigational medicinal product (IMP). Causes for death were metastases to central nervous system and viral infection.

**Table 6:** Serious Adverse Events in Cycles 1 or 2

	RGB-02 (N = 121) n (%)	Reference (N = 117) n (%)
Any Serious Adverse Event in Cycle 1 or 2	10 (8.3)	8 (6.8)
Blood and lymphatic system disorders	5 (4.1)	7 (6.0)
Febrile neutropenia	5 (4.1)	6 (5.1)
Neutropenia	0	2 (1.7)
Infections and infestations	2 (1.7)	1 (0.9)
Cystitis	0	1 (0.9)
Neutropenic infection	1 (0.8)	0
Oesophageal candidiasis	1 (0.8)	0
Vascular disorders	2 (1.7)	0
Lymphorrhoea	2 (1.7)	0
Gastrointestinal disorders	1 (0.8)	0
Haemorrhagic duodenitis	1 (0.8)	0
Erosive duodenitis	1 (0.8)	0
Neoplasms benign, malignant and unspecified (including cysts and polyps)	1 (0.8)	0
Metastases to central nervous system	1 (0.8)	0

### Immunogenicity

The screening assay test was negative in 96.1% of immunogenicity samples for the double blind treatment period. However, all positive screening tests were negative in the confirmatory test. Neutralising assay tests were not performed since none of the confirmatory tests were positive. In the open-label period including the 6-month follow - up, the screening assay test was negative in a range of 96.3–98.2% of all obtained immunogenicity samples at different time points. Again, positive screening tests turned out to be negative in the confirmatory test, therefore no neutralising assay tests were performed.

In conclusion, no patients of either treatment group had true positive immunogenicity results. The re-treatments did not increase the incidence of positive immune responses and the switch from reference treatment to RGB-02 did not provoke any immunogenic response.

## Discussion

This study was designed to confirm RGB-02 as a safe and effective biosimilar to the reference pegfilgrastim (Neulasta®) in a chemotherapeutic patient setting. The selected population received standard treatment with docetaxel/doxorubicin chemotherapy combination for breast cancer and was in close concordance to the populations chosen in former clinical trials with pegfilgrastim and biosimilar filgrastim trials. The chosen chemotherapy combination is known to cause severe neutropenia in almost all treated patients [22].

In this multinational, prospective, randomized double-blind study, therapeutic equivalence could be demonstrated between RGB-02 and the reference pegfilgrastim measuring the duration of severe neutropenia (DSN). The difference between treatments of 0.1 days and the 95% CI (− 0.2, 0.4) lies well inside the predefined range of ±1 day. The mean DSN observed in RGB-02 (1.7 ± 1.14 days) and the reference arm (1.6 ± 1.31 days) was in line with published data (mean DSN of 1.3–1.8 days) [23–27]. The analysis of secondary endpoints confirmed the positive findings for RGB-02. There was no clinically meaningful difference between treatment groups and the results showed similar evidence of benefit. The incidence and duration of severe neutropenia as well as episodes of fever and treatment with antibiotics were almost identical in both groups.

The safety results were comparable with published data for this chemotherapeutic regimen and pegfilgrastim [27, 28]. No new safety concerns were reported in this trial and RGB-02 was well tolerated with no related serious adverse events. Similarly, the incidence and severity of bone pain associated with the use of pegfilgrastim was comparable between treatment arms and was similar to previous studies [29].

Immunogenicity can cause problems for biologics. Patients may produce antidrug antibodies (ADAs), which might lead to efficacy loss or adverse reactions. No patients had true positive immunogenicity results for RGB-02 or the reference pegfilgrastim, i.e., no immunogenic response to the study drugs was observed during the study as no anti-pegfilgrastim antibodies were detected in the study.

Pegylated filgrastim when compared to filgrastim exerts a longer half-life and allows therefore a single dose per cycle thus increasing patient compliance and thereby reducing the risk of febrile neutropenic episodes.

## Conclusions

Therapeutic treatment equivalence between RGB-02 and Neulasta® was demonstrated. The analysis of the primary as well as all secondary efficacy endpoints did not reveal any statistically significant or clinically meaningful differences between the treatment arms. The safety profile of RGB-02 is comparable with the reference pegfilgrastim and no immunogenicity was found, even after switching from the originator product to RGB-02.

## Additional file

Additional file 1: List of Ethical Committees who approved the study RGB-02-101.(DOCX 12 kb)

## Abbreviations

ADA: Anti-drug antibody
AE: Adverse event
ANC: Absolute neutrophil count
ANCOVA: Analysis of covariance
BSA: Body surface area
CI: Confidence interval
CTCAE: Common terminology criteria for adverse events
DNS: Duration of severe neutropenia
ECG: Electrocardiogram
ECOG: Eastern cooperative oncology group
ESMO: European society for medical oncology
EU: European Union
EUDRACT: European union drug regulating authorities clinical trials
FAS: Full Analysis Set
GCP: Good Clinical Practice
G-CSF: Granulocyte-colony stimulating factor
ICH: International conference on harmonisation
IMP: Investigational medicinal product
IXRS: Interactive voice/web response system
LS: Mean least squares mean
PEG: Polyethylene glycol
PP: Per Protocol
SAE: Serious adverse event
std.: Standard deviation
TEAE: Treatment-emergent adverse event
ULN: Upper limit of normal
WBC: White blood cell
