# Supplementary material for: Efficacy and safety of RGB-02, a pegfilgrastim biosimilar to prevent chemotherapy-induced neutropenia: results of a randomized, double-blind phase III clinical study vs. reference pegfilgrastim in patients with breast cancer receiving chemotherapy
Source: BMC Cancer. 2019 Feb 6;19:122. doi: 10.1186/s12885-019-5329-6 (PMC6364429; doi:10.1186/s12885-019-5329-6)
Supplement: Supplementary file 1 — List of Ethical Committees who approved the study RGB-02-101.(DOCX 12 kb) [file 12885_2019_5329_MOESM1_ESM.docx]

**List of Ethical Committees who approved the study RGB-02-101:** Medical Research Council of Ethics Committee for Clinical Pharmacology, Hungary (Reg. No. 48676-0/2013-EKL), Ethics Committee on Clinical Trial on Human Medicinal Products, Ethics Committee of the Regional Hospital in Liberec, Czech Republic (Ref. No. EK/224/2013), Ethics Committee of Nemocnice Jindrichuv Hradec, a.s, Czech Republic, Ethics Committee of Na Bulovce Hospital, Czech Republic, Ethics Committee of Nemocnice Horovice, Czech Republic, Ethics Committee of Nemocnice Rudolfa a Stefanie Benesov a.s, Czech Republic, Agency for Medicinal Products and Medical Devices, Central Ethics Committee, Croatia (Reg. No. 381-15/60-13-02), Committee for Medicinal Products of General Hospital Pula, Croatia, Committee for Medicinal Products of Clinical Hospital Centre Zagreb, Croatia, Committee for Medicinal Products of Clinical Hospital Centre Osijek, Croatia, Ministry of Health, Ethics Committee for Multicentre Trials, Bulgaria (Ref. No. KI-292/21.12.2013), Ministry of Health, National Ethics Committee for the Clinical Study of Medicines, Romania (No. 3784; 4610), Ethical Council, Russia (Ref. No. 45596), Local Ethics Committee of State Budgetary Healthcare Institution of Stavropol Region Pyatigorsk Oncological Dispensary, Russia, Ethics Committee of AT N.N. Blokhin Russian Cancer Research Center of the Russian Academy of Medical Sciences, Russia, Ethics Committee of State Budgetary Healthcare Institution of Moscow, Moscow Municipal Oncology Hospital No. 62, Russia, Ethics Committee of State Autonomous Healthcare Institution (GAUZ) Republican Clinical Oncology Dispensary of the Ministry of Healthcare of the Republic of Tatarstan, Russia, Local Ethics Committee of Regional Budgetary Healthcare Institution Kursk Regional Clinical Oncological Dispensary, Russia, Ethics Committee of Ministry of Healthcare of Samara Region State Budgetary Healthcare Institution Samara Regional Clinical Oncology Dispensary (GBUZ SOCOD), Russia, Ethics Committee of State Budgetary Healthcare Institution of Moscow, Moscow Municipal Oncology Hospital No. 62, Russia, Ethics Committee of Archangelsk Clinical Oncology Dispensary, Russia, Center Ethics Committee of Municipal Institution of Health Protection, Kharkiv Regional Oncology, Ukraine (07 Mar 2014 No. 188 KD), Ethics Committee of Donetsk Regional Antitumor Center, Ukraine (No. 37/2014), Ethics Committee of Zakarpattya Regional Clinical Oncology Dispensary, Ukraine (26 Feb 2014), Ethics Committee of Regional Municipal Institution Sumy Regional Clinical Oncology Dispensary, Ukraine (03 Mar 2014 No. 51/4), Ethics Committee of Department of Healthcare of Uzhgorod City Council, Central City Clinical Hospital, Ukraine (04 Mar 2014 No. 97), Ethics Committee of Municipal Institution Dnipropetrovsk City Multifield Clinical Hospital #4 of Dnipropetrovsk Regional Council, Ukraine (13 Mar 2014), Ethics Committee of the Oncology Institute of Vojvodina, Sremska Kamenica, Serbia (No. 2058/p), Ethics Committee of Clinical Hospital Centre Zemun, Serbia (No. 2147/1), Ethics Committee of Clinical Center Kragujevac, Serbia (No. 01-12501), Ethics Committee of Clinical Hospital Center Bezanijska kosa, Serbia (No. 6317/3)
